# Risks and Benefits of Increased Nut Consumption: Cardiovascular Health Benefits Outweigh the Burden of Carcinogenic Effects Attributed to Aflatoxin B_1_ Exposure

**DOI:** 10.3390/nu9121355

**Published:** 2017-12-13

**Authors:** Hanna Eneroth, Stina Wallin, Karin Leander, Johan Nilsson Sommar, Agneta Åkesson

**Affiliations:** 1 National Food Agency, Box 622, 751 26 Uppsala, Sweden; stina.wallin@slv.se; 2 Institute of Environmental Medicine (IMM), Karolinska Institutet, Box 210, 171 77 Stockholm, Sweden; karin.leander@ki.se (K.L.), agneta.akesson@ki.se (A.Å.); 3 Department of Public Health and Clinical Medicine, Umeå University, 901 87 Umeå, Sweden; johan.sommar@umu.se

**Keywords:** nuts, healthy diet, risk-and benefit assessment, cardiovascular disease, myocardial infarction, stroke, aflatoxin, liver cancer, disability-adjusted life years

## Abstract

Nuts are rich in nutrients and mounting evidence shows that consumption reduces cardiovascular disease (CVD) incidence. Nuts may also be a major source of aflatoxin B_1_, a potent liver carcinogen and the risk/benefit balance is unknown. Based on national statistics and data from the PREDIMED intervention trial, we estimated the potential CVD-reduction if Swedes aged 55–79 consumed 30 g nuts/day, instead of the current national average of five grams per day. We also assessed the reduction in disability-adjusted life years (DALYs) due to myocardial infarction (MI) and stroke. We estimated the aflatoxin B_1_ exposure from nuts and calculated the margin of exposure. The approximation that one nanogram aflatoxin B_1_/kg body weight/day results in one additional liver cancer case/10 million person-years was used to estimate the number of liver cancer cases. The increased nut consumption scenario prevented more than 7000 CVDs in 2013 (306/100,000 person-years) and contributed to about 55,000 saved DALYs for stroke and 22,000 for MI. The concomitant increase in aflatoxin B_1_ exposure caused an estimated zero to three additional cases of liver cancer, corresponding to 159 DALYs spent, emphasizing the associated risks. Increased nut consumption, as part of a varied healthy diet, is warranted even when aflatoxin B_1_ exposure is taken into account. However, efforts to reduce aflatoxin exposure from food are essential.

## 1. Introduction

Evidence of the health benefits of nut consumption is increasing, with recent systematic reviews supporting the link between nut consumption and lower risk of coronary heart disease [1,2,3], cardiovascular disease (CVD) [1,4], and all-cause mortality [1,2,5]. Additional support for a causal relationship with CVD is provided by randomized clinical trials (RCTs) on the effects of nut consumption on blood lipid levels [6] and by the five-year PREDIMED intervention study performed in Spain among subjects at high CVD risk [7]. In the latter, a group consuming 30 g mixed nuts daily, in addition to their Mediterranean diet, had 28% lower risk of CVD compared with controls advised to adhere to a reduced-fat diet [7].

Although nuts are rich in polyunsaturated fatty acids (PUFA, mainly *n*-6), dietary fiber and micronutrients such as vitamin E, magnesium, and selenium, they may also contain high levels of one of the most potent carcinogenic substances, the mycotoxin aflatoxin B_1_ [8]. Aflatoxins are produced by molds and may be present in a range of foods, including nuts [9]. Both animal and epidemiological studies demonstrate associations between aflatoxin exposure and liver cancer [10], the second most common cause of death from cancer worldwide and a disease with a poor prognosis [11]. It has been estimated that 5–28% of the annual cases of hepatocellular carcinoma worldwide could be attributed to aflatoxin exposure [12], with the highest risk in regions with high aflatoxin exposure and high prevalence of chronic hepatitis B infection [10]. In European and other developed countries, cancer risk estimates are in the range <0.01–0.10 aflatoxin-induced cancers per 100,000 person-years, with wheat being the major contributing food commodity [13]. Nevertheless, because of its genotoxic carcinogenic properties no level of aflatoxin B_1_ exposure is considered safe, complicating any dietary advice or guidelines on nut consumption. To the best of our knowledge, the burden of liver cancer in relation to the CVD benefits of increased nut consumption has not been estimated previously.

The aim of the present study was to estimate the benefits and risks of increasing nut consumption to 30 g/day, following a proposed approach [14]. The calculations were based on the Swedish population, which has on average low (five grams per day) national nut consumption [15], a similar incidence of major CVDs as the European Union (EU) average [16], and a low incidence of liver cancer. We estimated the potential numbers of CVD cases avoided and the liver cancer cases attributable to the increased nut consumption. The absolute health impact was estimated through calculation of the burden of disability and death, expressed as disability-adjusted life years (DALYs).

## 2. Materials and Methods

### 2.1. Setting

The Swedish population in 2013 (9.6 million inhabitants [17]) had an intermediate burden of CVD compared with other European countries, corresponding of 60 DALYs/1000 individuals [18]. Although CVD prevalence in Western Europe is low by global standards, CVD is the most common cause of death and disability [19]. The incidence of liver cancer in Sweden is low: 3.7/100,000 person-years for women and 8.7/100,000 person-years for men [20]. Nut consumption, based on a nation-wide dietary survey, is on average 5 g/day for women and men (women, *n* = 1005, mean 5 g, standard deviation 12 g; men, *n* = 792, mean 4 g standard deviation, 13 g/day) [15].

### 2.2. Change in Nutrient Intake

To estimate the changes in nutrient intake brought about by increased consumption of nuts in the population, we used a scenario with iso-caloric replacement of part of the average diet of adults in the nation-wide dietary survey [15]. Substitution of nuts for “average intake” was chosen because it is difficult to assume how increased nut intake would change the diet. The assumed consumption of 30 g/day of the PREDIMED mixture of nuts (15 g walnuts, 7.5 g hazelnuts and 7.5 g almonds [7]), corresponded to 10% (817 kJ) of the total energy intake reported.

### 2.3. Estimating Benefit: Nut Consumption and Cardiovascular Disease

To estimate the CVD benefits in this study, we applied a scenario using the observed effects for the primary and secondary endpoints in the nut intervention in PREDIMED (30 g/day) on CVD incidence in the population. The primary endpoint was composite CVD, defined as fatal and non-fatal MI and stroke and death from heart failure, cardiac arrest, cardiomyopathy, pulmonary embolism, and aortic aneurysm, with hazard ratio (HR) 0.72 (95% confidence interval (CI) 0.54–0.96) [7]. Three secondary endpoints related to CVD were also tested in the PREDIMED [7]:

1. fatal and non-fatal MI; HR 0.74 (95% CI 0.46–1.19),
2. fatal and non-fatal stroke;HR 0.54 (95% CI 0.35–0.84), and
3. CVD mortality, comprising CVD death other than MI and stroke (not affected by the nut intervention; HR 1.01, 95% CI 0.61–1.66).

We then obtained data on the total number of first-incident fatal and non-fatal MI and stroke in the population in 2013 (after seven disease-free years), and deaths from the other CVDs, from the Patient Register and Cause of Death Register (National Board of Health and Welfare). To match the PREDIMED study, we focused on the age group 55- to 79-year-old women and men.

Based on the HRs reported in the PREDIMED trial as stated above and the assumption that the population increased nut consumption from on average 5 to 30 g/day individual consumption, we estimated:

- the number of incident composite CVD, MI, and stroke events that could potentially be prevented ((1-HR) times the number of events in the population) and
- the corresponding reduction in disease burden, expressed as DALYs due to fatal and non-fatal MI and stroke.

We did not include the secondary endpoint CVD deaths (other than MI or stroke) as a separate outcome in the assessment of DALYs. DALYs were calculated as the sum of years lost to disability (YLD) and years of life lost (YLL) as a result of pre-term mortality due to MI and stroke, where

YLL = N × L(1)

N = mortality rates, L = life expectancy at obtained age. And

YLD = I × DW × L(2)

I = incidence, DW = disability weight, L = expected duration of disability. The DW factors used were 0.439 for non-fatal cases of MI and DW = 0.266 for stroke [21]. The long-term survival time after MI was obtained by assuming that the mortality rate in each age group was twice as high after MI as the average mortality rate in that age group [22]. Corresponding data for stroke cases were obtained from Eriksson et al., who estimated the long-term survival after a first stroke, from which a straight-line extrapolation of survival was made after the end of study follow-up [23].

### 2.4. Estimating Risk: Aflatoxin Exposure and Liver Cancer

To predict the risk, we estimated the average aflatoxin B_1_ exposure via nuts in the study population and compared the exposure to existing risk assessments for liver cancer [10]. We used the mean aflatoxin B_1_ concentrations obtained for the various nut types, sampled within the EU during the period 2000–2006 [8], to estimate aflatoxin B_1_ exposure at the current population average consumption (5 g nuts/day) [15] and in the scenario of 30 g nuts/day. Although there are several aflatoxin congeners (e.g., B_1_, G_1_, M_1_, B_2_, G_2_), most of the available toxicological data relate to aflatoxin B_1_ and its relative potency is highest (B_1_ > (G_1_, M_1_) >> (B_2_, G_2_)). Moreover, B_1_ is the most frequent aflatoxin present in contaminated samples, whereas B_2_, G_1_, and G_2_ are generally not reported in the absence of B_1_ [8]. The levels of aflatoxin B_1_ vary considerably both within and between different types of nuts, as is apparent in terms of both number of samples with detectable levels and actual levels observed. No detectable levels (i.e., concentrations below the limit of detection) are observed in 70–90% of samples of almonds, hazelnuts, peanuts, cashews, and “other nuts” analyzed in the EU, while the corresponding proportion of non-detectable levels for both pistachios and Brazil nuts is only 56% [7]. The highest concentrations of aflatoxin B_1_ are also detected in these two nut types [7]. In order not to underestimate the exposure, non-detection of aflatoxin B_1_ concentrations was set at the limit of detection for each nut type (≈0.1–0.2 µg/kg) and subsequently used to calculate the mean contamination level in nuts. This resulted in an average of 6.4 µg aflatoxin B_1_/kg nuts for all nut types, while excluding pistachios and Brazil nuts resulted in an average of 1.2 µg/kg. Therefore, we performed separate intake estimations for the group of all nuts and for nuts excluding pistachios and Brazil nuts.

Based on epidemiological data and a model average from the different statistical models used, the Joint FAO/WHO Expert Committee on Food Additives (JECFA) estimates that aflatoxin B_1_ exposure of 1 ng/kg body weight (bw) and day results in roughly one additional case of liver cancer per 10 million individuals each year in a population of non-carriers of hepatitis B [10]. This impact was considered independent on the background liver cancer rate. We used this reference point to assess the number of liver cancer cases expected to occur due to increased nut consumption. Data on the number of primary and unspecified liver cancer cases occurring in the Swedish population in 2013 were obtained from the Swedish Cancer Register [24].

Because tolerable daily intake (TDI) is not meaningful to derive for genotoxins, an alternative approach, the margin of exposure (MOE), is used as a complement to the reference dose by JECFA [25]. Thus, we complemented the estimates of expected number of liver cancer cases with estimates of MOE.

MOE = benchmark dose lower limit (BMDL)/average population exposure in ng/kg bw(3)

The benchmark dose (BMD) is a modeling of available data from either animal or epidemiological studies and an extrapolation of the exposure level that would cause a certain predefined increase in cancer incidence relative to the baseline incidence. The BMDL refers to the lower limit of the CI, which takes into account the uncertainty inherent in the underlying studies, assuring with 95% confidence that the chosen adverse response is not exceeded. For aflatoxin B_1_, the European Food Safety Authority (EFSA) has derived BMDL values based both on animal studies and on epidemiological data [8]. From animal data, the BMDL_10_ (10% higher incidence of cancer relative to baseline) is set to 170 ng/kg bw/day. From human data, the BMDL_10_ is set to 870 ng/kg bw/day and BMDL_1_ (1% higher incidence of cancer relative to baseline) to 78 ng/kg bw/day [8]. As a rule of thumb, a MOE of 10,000 or more based on data from animal studies indicates exposure of low public health concern.

Because the estimates of both dietary aflatoxin B_1_ exposure and number of liver cancer cases attributable to the exposure were based on several assumptions, detailed assessment of DALYs from liver cancer was not meaningful. Instead, we simplified the DALY estimations by assuming that, irrespective of sex, all liver cancer diagnoses occurred at age 30 years and that all had immediate fatal outcome (DALYs = YLL), with a remaining life expectancy of 53 years [26]. The rationale for using age 30 years was to avoid underestimation of DALYs compared with using the average age at diagnosis. Liver cancer is rare at a young age and the likelihood of a link to lifestyle factors such as nut consumption is lower.

The analyses presented in this paper are scenarios with published data from the PREDIMED trial and publicly available national data. Thus, no data collection was performed and therefore ethical approval and informed consent are not applicable for this study.

## 3. Results

The modeled change in iso-caloric replacement of part of the average diet with 30 g nuts of the PREDIMED mix resulted in an average 50% increase in PUFA and 20% increase in monounsaturated fatty acid (MUFA) intake (Table A1). Intake of vitamin E and magnesium was also increased.

For the primary endpoint, the composite CVD (including fatal and non-fatal MI and stroke, and other CVD deaths), 27,428 cases (1094/100,000 person-years) occurred in 55- to 79-year-olds in Sweden 2013. The 28% lower risk (95% CI 4–46%) obtained for this primary outcome in the group supplemented with 30 g of nuts/day in PREDIMED would thus result in 7680 prevented cases (95% CI 1097–12,617), corresponding to 306/100,000 person-years. The estimated number of preventable cases of MI and stroke, corresponding to the two secondary endpoints, was 3715 (95% CI 0–7715) and 5390 (95% CI 1875–7617) respectively (Table 1 and Table 2).

The absolute health impact, expressed as DALYs gained by increasing current nut consumption to 30 g/day, was about 22,000 for MI (Table 1) and 55,000 for stroke (Table 2) in women and men combined. Most of the DALYs were attributed to mortality, especially for MI, where time lived with disability was expected to be short. The higher incidence of MI and stroke among men than among women was reflected in higher DALYs for men.

As shown in Table 3, in the scenario where Brazil nuts and pistachios were excluded from consumption, the estimated exposure to aflatoxin B_1_ from nuts (0.4–0.5 ng/kg bw and day) did not reach the exposure level of 1 ng/kg bw and day, resulting in no additional cases of liver cancer in the population (Table 3). The corresponding calculation based on a nut mixture that included Brazil nuts and pistachios resulted in aflatoxin B_1_ exposure of 2.3–2.8 ng/kg bw and day, which may correspond to three additional cases of liver cancer each year in Sweden. On the other hand, the estimated MOE, 60–400 based on animal data, indicated a public health concern in both exposure scenarios, since the margin of 10,000 was not met. The scenario of three additional cases of liver cancer at age of 30 years, no disability, and with a fatal outcome resulted in an estimated 159 DALYs from liver cancer associated with nut consumption.

## 4. Discussion

In the population of Swedish residents aged 55–79 years in 2013, with an average low exposure to aflatoxins, increasing the nut consumption from the current average of five grams per day to individual consumption of 30 g/day could prevent 7680 individuals from developing a first CVD (306/100,000 person-year) and could contribute about 55,000 saved DALYs for stroke and 22,000 for MI. At the same time, the potential increase in aflatoxin B_1_ exposure would lead to an estimated zero to three additional cases of liver cancer in the population, corresponding to approximately 159 DALYs. Thus, the population health benefits provided by increased nut consumption clearly outweigh the risks associated with increased aflatoxin B_1_ exposure.

The combined epidemiological evidence [1,2,4] and results from RCTs [6,7] provide unusually strong support for positive health effects of nuts, with the group “nuts and seeds” being graded high on most criteria for causality in prevention of ischemic heart disease [4]. This despite the fact that observational data indicate that nut eaters generally have a healthier lifestyle than people who never or rarely consume nuts [27,28]. Moreover, the association between nut consumption and CVD health is evident in a range of populations, despite imprecise assessments of dose and types [2].

The use of RCT data was supported by a similar incidence of ischemic heart disease in the studied Swedish residents as in the trial population, despite the fact that the PREDIMED subjects were chosen to be at higher risk of CVD. In any case, the risk estimates were similar in the RCT and in the summary of observational studies: for CVD the relative risk (RR) was 0.79 (95% CI 0.70–0.88)/28 g/day serving [1], compared with HR 0.72 for composite CVD in the group consuming 30 g nuts/day in the PREDIMED RCT [7]. In contrast to the RCT, recent meta-analyses do not show any significant associations between nut intake and risk of stroke, either as total (RR 0.93; 95% CI 0.83–1.05) [1], ischemic (RR 1.06; 95% CI 0.81–1.38) [2], or stroke mortality (RR 0.83; 95% CI 0.69–1.00) [2]. Indications of an inverse association with stroke incidence up to an intake of 15–20 g nuts/day (non-linear association) [1] and with intake of peanuts [1,29] and walnuts [29] have been observed, but a recent evaluation graded the evidence for stroke as very low [2]. A potential counteracting effect of salt, often added to nuts (but not in the PREDIMED trial), on stroke risk cannot be excluded.

The favorable fat composition of nuts and the content of micronutrients, bioactive compounds, and dietary fiber may contribute to lower CVD by improving the cardio-metabolic profile [6]. Increased nut consumption is one way to increase adherence to the dietary recommendations on fat intake, where roughly two-thirds should comprise unsaturated fats [30,31]. A 30 g portion of nuts (about a handful, corresponding to ~10% of total energy intake), compared with the low current average Swedish nut consumption, can be considered a major dietary change. However, a recent meta-analysis indicated that smaller amounts are also associated with health benefits [1]. Dose-response data indicate lower risk for coronary heart disease with increasing nut consumption up to about 10–15 g/day [3] and association with reductions in all-cause mortality up to 15–20 g/day [5]. Micha et al. concluded that five servings/week, each of about 28 g, is the optimal mean population intake based on ischemic heart disease prevention [4].

A MOE of 10,000 or higher, based on BMDL_10_ from an animal study, is suggested to be a low public health concern [25], but was not met even at the current level of nut consumption. At present, there is no alternative margin proposed for data based on epidemiological studies in humans. The MOEs obtained from animal data after increased consumption are substantially lower (60–400) than the desirable margin, however, well agreeing with our estimate of zero to three additional cases of liver cancer in the total population. Focusing on aflatoxin B_1_ exposure alone, increased nut consumption is clearly of public health concern, while broadening the picture to include beneficial effects of nuts on CVDs indicates that many more lives are saved than lost.

We focused on CVD and liver cancer alone, although nut consumption has been associated with reduced all-cause mortality [1,2,5] and type 2 diabetes [4,32] and improved weight maintenance [33,34], but also with exposure to salmonella [35] and acrylamide [35]. Prevention of premature deaths would have a large impact on the population, but we chose not to include this imprecise outcome in our assessment. The effect on weight maintenance and type 2 diabetes could be of public health importance, but data on effect size are not as certain as for CVD. Risk of salmonellosis was judged to be limited, as was the risk associated with acrylamide exposure from heat-treated nuts [35]. Allergy to peanuts and tree nuts, which is common in Sweden, is another potential health concern. People who are allergic to nuts may have greater difficulties avoiding nuts if overall consumption increases, an effect we did not account for in our scenario. On the other hand, measures to prevent allergy are currently under discussion and avoidance of highly allergenic foods such as peanuts is no longer the preferred strategy for allergy prevention [36].

The present study is one of only a few to weigh health benefits against risks of foods using the burden of disease as an outcome and is unique in doing so for nuts, which has implications for dietary guidelines worldwide. One strength of our study is the fact that the assessment of CVD benefits was based on risk estimates from an RCT, providing the highest grade of evidence and lowering the risk of bias due to, e.g., confounding effects. The availability of reliable data on morbidity and mortality in the Swedish population [37,38] was also a strength.

There are important limitations of our study. First, and most importantly, our results are conditional on consumption of nuts and their impact on CVD incidence in our study population resembling those in the PREDIMED study. Obviously, the effect of increased nut consumption in our studied population, with a dietary pattern different from the Mediterranean diet, is unknown. Thus, the estimated number of prevented cases needs to be considered tentative in a public health context of CVD prevention, although still justified and valid for the purpose of our risk-benefit comparison. Importantly, our results were robust in the sense that the conclusion prevails even if the part of CVD reduction attributed to nuts would be considerably lower. The results are also conditional on the assumption that there was no net increase in energy intake altering any risk factor profile in the population and that aflatoxin B_1_ concentration in nuts sampled within the EU is representative of the exposure in the target population. The varying aflatoxin B_1_ concentrations present in nuts clearly introduce uncertainties, and we did not assess any adverse effect of exceeding the target consumption of 30 g/day.

Second, for comparing different disease outcomes we used DALYs despite the uncertainty that follows with making the necessary assumptions. DALYs is the most accepted measure for comparing disease and risk factors and used in the Global Burden of Disease project [39]. Because register information was not cross-linked in the present study, we could not exclude the possibility that some individuals with incident non-fatal MI or stroke were counted more than once, if they either died from CVD other than MI or stroke in the same year (2013) or encountered both MI and stroke in that year. This may have slightly overestimated the number of prevented cases of composite CVDs. Similarly, we could not exclude the possibility that individuals who suffered from both incident non-fatal MI and stroke during 2013 were counted twice in the estimation of DALYs. However, considering the relatively short period of disability for MI, the subsequent overestimation of DALYs is likely minor. Furthermore, data on proportions with specific disabilities among the stroke cases in our population were not available, preventing us from sub-classifying stroke cases according to the most recent DWs [40], and thus we used composite DWs from 2004 [21]. This could have influenced our estimated DALYs in any direction.

Third, the lack of data on the potential health impact of aflatoxin exposure in a low-exposure population with a low prevalence of hepatitis [41,42] introduces large uncertainties in estimation of adverse health impacts. Important risk factors for liver cancer are alcohol consumption and chronic hepatitis B infection [8], while aflatoxin is considered unlikely to be a major contributor to liver cancer in the Nordic countries. The JECFA potency estimate is derived from human studies limited to populations with a high prevalence of hepatitis B infection and with very limited information on aflatoxin B_1_ exposure. However, even an unrealistic assumption that all liver cancer in Sweden was due to aflatoxin B_1_ exposure would not change the conclusion that the CVD reductions outweigh the burden of liver cancer.

Altogether, our study provides an example of how different health aspects of a type of food can be included in the same evaluation. Comparing the most pertinent health benefits and potential risks associated with nut consumption, provides strong support for increasing overall nut consumption and for the inclusion of nuts in dietary guidelines. In policymaking, considerations including the environmental impact of nut production, population dietary habits, disease pattern, and allergy prevalence may be necessary.

Future research should focus on the amount and type of nuts for optimal benefits and more resources should be allocated to monitoring the exposure and health impact of aflatoxins in food. Because the intake of aflatoxins from single nuts with high concentrations of aflatoxins is potentially more influential for exposure than the total amount of nuts consumed, the most important measure to limit exposure is to prevent nuts with high aflatoxin concentration from reaching consumers. Improvements in production, storage, and control are vital to ensure that nuts are part of a safe, nutritious diet.

## 5. Conclusions

In a scenario with national data on exposure and outcome, combined with estimates from a RCT, we showed that health benefits, measured as cases of CVD potentially averted and DALYs saved, outweighed any additional cases of liver cancer due to increased nut consumption. Our conclusion is that increased nut consumption, as part of a varied healthy diet, is warranted even when aflatoxin B_1_ exposure is taken into account. However, efforts to reduce aflatoxin exposure from nuts are essential.

## Figures and Tables

**Table 1:** Estimated absolute gain in health impact attributable to first incident MI in the Swedish population aged 55–79 years in 2013, by increasing the nut consumption from current average of 5 g/day to a scenario where everyone consumes 30 g/day.

	Current Average Intake (5 g/Day)					At 30 g Nuts/Day ^2^			Gain			
	I	No of Cases ^1^	YLL	YLD ^3^	DALYs	YLL	YLD ^3^	DALYs	Prevented Cases	YLL	YLD ^3^	DALYs
Women												
55–59	137	394	2672	136	2807	1977	100	2077	102	695	35	730
60–64	189	549	3366	166	3533	2491	123	2614	143	875	43	918
65–69	293	886	5256	251	5507	3890	186	4075	230	1367	65	1432
70–74	504	1128	7416	347	7763	5488	257	5745	293	1928	90	2018
75–79	787	1350	7777	349	8126	5755	259	6013	351	2022	91	2113
All ages	338	4307	26,487	1249	27,737	19,601	924	20,525	1120	6887	325	7211
Men												
55–59	426	1232	7026	410	7436	5199	304	5503	320	1827	107	1933
60–64	618	1778	10,334	515	10,848	7647	381	8028	462	2687	134	2821
65–69	803	2388	14,688	659	15,346	10,869	487	11,356	621	3819	171	3990
70–74	1,126	2387	13,360	631	13,991	9887	467	10,353	621	3474	164	3638
75–79	1,510	2195	10,338	541	10,879	7650	400	8050	571	2688	141	2828
All ages	810	9980	55,746	2755	58,501	41,252	2039	43,291	2595	14,494	716	15,210
Total	683	14,287	82,233	4004	86,238	60,853	2963	63,816	3715	21,381	1041	22,421

Abbreviations: MI, myocardial infarction; I, incidence; YLL, years of life lost; YLD, years lost to disability; DALYs, disability-adjusted life years. ^1^ Number of MI in 2013. ^2^ Based on a reduced risk of HR 0.74 by increased consumption of walnuts, hazelnuts, and almonds [6] in a scenario where everyone in the population consumes 30 g/day. ^3^ Disability weight 0.439 [18].

**Table 2:** Estimated absolute gain in health impact attributable to first incident stroke in the Swedish population aged 55–79 years in 2013, by increasing the nut consumption from current average of 5 g/day to a scenario where everyone consumes 30 g/day.

	Current Average Intake (5 g/Day)					At 30 g Nuts/Day ^2^			Gain			
	I	No of Cases ^1^	YLL	YLD ^3^	DALYs	YLL	YLD ^3^	DALYs	Prevented Cases	YLL	YLD ^3^	DALYs
Women												
55–59	121	346	5834	886	6719	3150	478	3629	159	2684	407	3091
60–64	194	561	7288	1326	8614	3935	716	4652	258	3352	610	3962
65–69	320	968	9025	2227	11,252	4874	1203	6076	445	4152	1024	5176
70–74	542	1211	9531	2162	11,693	5147	1168	6314	557	4384	995	5379
75–79	906	1554	9453	1990	11,444	5105	1075	6180	715	4348	916	5264
All ages	364	4640	41,131	8591	49,722	22,211	4639	26,850	2134	18,920	3952	22,872
Men												
55–59	225	651	11,008	1228	12,236	5944	663	6607	299	5064	565	5629
60–64	378	1089	13,519	2053	15,572	7300	1108	8409	501	6219	944	7163
65–69	545	1619	12,590	3393	15,983	6799	1832	8631	745	5791	1561	7352
70–74	864	1832	11,763	2972	14,736	6352	1605	7957	843	5411	1367	6778
75–79	1291	1887	9140	2202	11,342	4935	1189	6125	868	4204	1013	5217
All ages	1291	7078	58,020	11,849	69,869	31,331	6398	37,729	3256	26,689	5450	32,140
Total	467	11,718	99,151	20,440	119,591	53,542	11,037	64,579	5390	45,609	9402	55,012

Abbreviations: I, incidence; YLL, years of life lost; YLD, years lost to disability; DALYs, disability-adjusted life years. ^1^ Number of stroke cases in 2013. ^2^ Based on a reduced risk of HR 0.54 by increased consumption of walnuts, hazelnuts, and almonds [6] in a scenario where everyone in the population consumes 30 g/day. ^3^ Disability weight 0.266 [18].

**Table 3:** Aflatoxin B_1_ exposure and margin of exposure (MOE) by sex and level of nut consumption.

	All Nuts ^1^		All Nuts Excluding Brazil Nuts and Pistachios ^2^	
Nut consumption (g/day)	5	30	5	30
Women, 69 kg				
Aflatoxin B_1_ exposure, ng/kg bw day	0.5	2.8	0.08	0.5
MOE, animal data ^3^	370	60	2100	350
MOE, human data ^4^	1900	310	10,700	1800
Men, 84 kg				
Aflatoxin B_1_ exposure, ng/kg bw day	0.4	2.3	0.07	0.4
MOE, animal data ^3^	445	75	2400	402
MOE, human data ^4^	2300	380	12,300	2200

^1^ Mean aflatoxin B_1_ concentration of 6.4 µg/kg nut for cashews, hazelnuts, peanuts, almonds, pistachios, Brazil nuts, and “other nuts” sampled in the EU 2000–2006 [8]. ^2^ Mean aflatoxin B_1_ concentration of 1.2 µg/kg nut for cashews, hazelnuts, peanuts, almonds, and “other nuts” sampled in the EU 2000–2006 [8]. ^3^ Based on benchmark dose lower limit (BMDL)_10_ of 170 ng/kg bw day from animal data. ^4^ Based on BMDL_10_ of 870 ng/kg bw day from human data.

## Appendix A

**Table A1:** Changes in daily nutrient intakes in a scenario where the energy content of the PREDIMED nut mix 817 kJ replaces the corresponding amount of the average diet in men and women from a nation-wide dietary survey [15].

	Women (1005)		Men (792)	
	Current Intake	Change, %	Current Intake	Change, %
Energy, kJ	7421	0	9359	0
SFA, g	27	−5	33	−4
MUFA, g	26	21	33	17
PUFA, g	12	52	14	43
Vitamin E, mg	10	26	11	25
Folate, µg	253	−3	266	−1
Calcium, mg	820	−5	944	−4
Magnesium, mg	305	17	364	15
Selenium, µg	42	−9	50	−7
Zinc, mg	10	−2	12	−2

Abbreviations: SFA, saturated fatty acids; MUFA, monounsaturated fatty acids; PUFA, polyunsaturated fatty acids. Nutrient content from National Food Agency Food Database [43].
